# Alternative life‐history strategy contributions to effective population size in a naturally spawning salmon population

**DOI:** 10.1111/eva.13580

**Published:** 2023-07-14

**Authors:** Erika King, Megan V. McPhee, Scott C. Vulstek, Curry J. Cunningham, Joshua R. Russell, David A. Tallmon

**Affiliations:** ^1^ College of Fisheries and Ocean Sciences University of Alaska Fairbanks Alaska USA; ^2^ National Oceanic and Atmospheric Administration Juneau Alaska USA; ^3^ Biology and Marine Biology Program University of Alaska Southeast Juneau Alaska USA

**Keywords:** effective number of breeders, effective population size, life history, salmonids, SNPs

## Abstract

Alternative life‐history tactics are predicted to affect within‐population genetic processes but have received little attention. For example, the impact of precocious males on effective population size (*N*_e_) has not been quantified directly in Pacific salmon *Oncorhynchus* spp., even though they can make up a large percentage of the total male spawners. We investigated the contribution of precocial males (“jacks”) to *N*_e_ in a naturally spawning population of Coho Salmon *O. kisutch* from the Auke Creek watershed in Juneau, Alaska. Mature adults that returned from 2009 to 2019 (~8000 individuals) were genotyped at 259 single‐nucleotide polymorphism (SNP) loci for parentage analysis. We used demographic and genetic methods to estimate the effective number of breeders per year (*N*_b_). Jack contribution to *N*_b_ was assessed by comparing values of *N*_b_ calculated with and without jacks and their offspring. Over a range of *N*_b_ values (108–406), the average jack contribution to *N*_b_ from 2009 to 2015 was 12.9% (SE = 3.8%). Jacks consistently made up over 20% of the total male spawners. The presence of jacks did not seem to influence *N*_b_/*N*. The linkage disequilibrium *N*_e_ estimate was lower than the demographic estimate, possibly due to immigration effects on population genetic processes: based on external marks and parentage data, we estimated that immigrant spawners produced 4.5% of all returning offspring. Our results demonstrate that jacks can influence *N*_b_ and *N*_e_ and can make a substantial contribution to population dynamics and conservation of threatened stocks.

## 1 INTRODUCTION

Effective population size (*N*_e_) and effective number of breeders (*N*_b_) are important parameters in evolutionary and conservation biology because they influence the impacts of selection, migration, inbreeding, and genetic drift. In turn, *N*_e_ and *N*_b_ are affected by variation in survival and reproductive rates associated with alternative life histories (Waples, 2006a; Waples et al., 2011, 2013, 2018; Waples & Antao, 2014). Theory predicts that any of the many different morphological, behavioral, and age‐specific traits that change variance in reproductive success among individuals can translate into changes in *N*_e_ and *N*_b_ (Crow & Kimura, 1970). A survey of a diverse array of animal and plant life histories showed that age‐at‐maturity and adult lifespan, two life‐history parameters that impact variance in reproductive success, can dramatically affect variation in *N*_e_ and *N*_b_ and *N*_b_/*N*_e_ (Waples et al., 2013).

Despite a rich theoretical framework to incorporate life‐history variation into *N*_e_ and *N*_b_ calculations (Charlesworth, 1994; Wang et al., 2016), alternative and cryptic life histories are often overlooked because they are difficult to study and complicate analyses (but see Johnstone et al., 2013; Perrier et al., 2014; Saura et al., 2008). Yet, life‐history variation is ubiquitous across species, and different life‐history strategies impact a variety of critically important population processes and parameters (Charlesworth, 1994; Den Boer, 1968). For example, studies of semelparous plants and animals have shown that variable age‐at‐reproduction can increase population viability (Acker et al., 2014), population growth rate (Greene et al., 2010), and *N*_e_ (Watters et al., 2003). Similarly, immigrants can profoundly influence local adaptation (Lenormand, 2002) and increase population persistence over time (Hill et al., 2002). Consequently, empirical studies where the effects of life‐history variation on *N*_b_ and *N*_e_ are quantified over multiple generations are particularly valuable.

Fishes are known for their diverse life‐history and mating strategies (Avise et al., 2002), and the family Salmonidae has particularly unique and well‐studied strategies. Salmonids lay their eggs in riverbeds, after the eggs hatch, individuals rear in freshwater until they out‐migrate to brackish waters or the sea for better growth opportunities, and after this most migrate back to their river of origin to spawn. Atlantic salmon (*Salmo salar*) and several species of Pacific salmon (*Oncorhynchus* spp.) exhibit alternative life‐history tactics: some males reach sexual maturity at smaller size and younger age compared to typical full‐sized males that mature after gaining more mass after spending more time in the ocean (Gross, 1985). In Atlantic salmon, males that mature without migrating to sea are referred to as “mature male parr.” In populations of Coho, Sockeye, and Chinook Salmon, anadromous males that return to spawn after less time at sea than full‐size males are referred to as “jacks.” Jacks and mature male parr can outnumber full‐sized males during reproduction, but their relative abundance varies greatly across species, populations, and years.

Although most genetic studies of closely related Atlantic Salmon do not explicitly consider mature male parr in *N*_e_ and *N*_b_ estimates because they are difficult to sample (Ferchaud et al., 2016), where their contributions have been quantified (e.g., Perrier et al., 2014; Saura et al., 2008) or inferred (Johnstone et al., 2013), they substantially increase *N*_b_ and *N*_e_. Saura et al. (2008) found mature male parr decrease variance in male reproductive success, have little effect on the reproductive success of females, and increase overall *N*_e_ by 2–3 times without affecting the *N*_e_/*N* ratio. Similarly, Perrier et al. (2014) found mature male parr increase *N*_b_ by roughly two‐fold, even though they had lower reproductive success. In contrast, mature male parr contributions to *N*_e_ have been much smaller in experimental (Garcia‐Vazquez et al., 2001; Jones & Hutchings, 2001, 2002) and simulation‐based (Palstra et al., 2009) studies of Atlantic Salmon. Based on these results, we predicted that jacks would at least somewhat increase Auke Creek Coho Salmon *N*_e_ and *N*_b_.

In some Pacific salmon species, jacks spend reduced time in saltwater and can make up anywhere from <1 (Sockeye Salmon *O. nerka*; Quinn et al., 2001) to >50% of returning males (Coho Salmon *O. kisutch*; King et al., 2023). Observational studies show that jacks participate in spawning (Gross, 1985; Healey & Prince, 1998). In experimental settings, Sockeye Salmon jacks sired 3%–93% of eggs fertilized per female (Foote et al., 1997), and jacks sired 20% of total fry offspring of Chinook Salmon (*O. tshawytcha*; Berejikian et al., 2010). These data suggest the alternative jack life history can contribute significantly to population genetic processes in wild Pacific salmon populations. Yet, their contributions to *N*_e_ in natural populations are poorly understood, probably because they are more difficult to detect and count accurately due to their small size and because of their small economic value relative to full‐sized individuals. To our knowledge, there has been no multi‐generational study to quantify jack contribution to *N*_b_ and *N*_e_ using individual adult‐to‐adult reproductive success from all individuals in a population of Pacific salmon. It is important to understand the effect of variable life histories on *N*_b_ and *N*_e_ because such variability can further complicate the relationship between census size and effective population size, complicating management for long‐term population viability.

Besides diverse male mating tactics, another defining feature of Pacific salmon life history is the return of most adults to their natal stream to spawn, termed “homing.” Homing of adults to their natal stream facilitates adaptation to local spawning conditions, but some individuals fail to return home, instead “straying” into other spawning streams. For a given population, reproductively successful strays from other streams are effectively immigrants that add adaptive, maladaptive, or neutral genetic variation and will affect population genetic processes over time. As straying increases, local genetic processes will increasingly reflect regional processes and effective sizes (Kimura & Maruyama, 1971; Palstra & Ruzzante, 2008; Ryman et al., 2019). However, estimates of the frequency and reproductive success of strays vary greatly among salmon species, regions, and other factors, and are poorly understood and rarely quantified in wild populations.

We used a long‐term and detailed demographic and genetic dataset to determine the contribution of jacks to the effective number of breeders and effective population size over multiple generations in a naturally spawning population of Pacific salmon in Southeast Alaska. To achieve this, we quantified adult‐to‐adult reproductive success of Auke Creek coho salmon over 11 years. We determined the percentage of successful reproducers that had strayed into this population, based on comprehensive genetic sampling of returning adults and marking of out‐migrating smolts (juveniles switching to marine habitats). Our specific objectives were to (i) quantify the contribution of jacks to *N*_b_ and *N*_e_ using demographic and genetic methods; (ii) compare *N*_b_ and *N*_e_ estimates among methods; (iii) estimate age‐specific contributions to *N*_b_ and *N*_e_ using life tables, and (iv) quantify the reproductive success of immigrants into this population. We found that jack life history contributes substantially to *N*_b_ and *N*_e_. Immigrants were among the adults that reproduced successfully and their contribution influenced *N*_e_ and likely the population genetic dynamics of the population. We discuss the implications of our findings for small and declining populations of Pacific salmon.

## 2 MATERIALS AND METHODS

### 2.1 Study population

This study was conducted on the Coho Salmon population in the Auke Lake drainage in Juneau, Alaska, USA. Tissue samples and demographic information were collected at a permanent two‐way weir located between Auke Lake and Auke Bay that is operated by the National Oceanic and Atmospheric Administration (NOAA). Counts of out‐migrating smolts and returning adults have been recorded annually since 1980 and genetic samples have been collected since 2009. The weir catches all out‐migrating smolts and the smolts are released downstream after their adipose fin is clipped and they receive a coded‐wire tag. The weir also captures all returning adults, which can be identified as originating from Auke Creek by the absence of an adipose fin. As each adult is manually transported over the weir, an axillary process is removed and stored in 95% ethanol and each individual is assigned a sex/spawning type (female, full‐size male, or jack) based on morphological characteristics. Roughly one‐third of returning adults are sampled for length and age. Adults with intact adipose fins could either be adults that strayed into Auke Creek from other populations or were smolts that avoided capture and tagging at the weir; we used parentage (described below) to distinguish between these two possibilities. This study did not require ethics approval because we exclusively analyzed archived tissue samples collected by NOAA and not live fish. This study used data from adults returned between 2009 and 2019. Auke Creek Coho Salmon eggs are laid in the fall and after hatching in the spring, individuals spend either 1 or 2 years rearing in freshwater before smolting and migrating to sea. Jacks spend 6 months and full‐size females and males spend 1 full year at sea before returning to spawn (Table 1). Females and full‐sized males return 3 or 4 years after being spawned (this includes time spent in the gravel, 1 or 2 years in freshwater, 1 year at sea, and returning to spawn). Jacks return 2 or 3 years after being spawned (this includes time spent in the gravel, 1 or 2 years in freshwater, 6 months at sea, and returning to spawn). Males that return after 6 months at sea, regardless of freshwater age, are referred to as jacks and are clearly smaller than males that spent a full year at sea. Age classes are abbreviated as 1.0 (2‐year‐old jack), 1.1 (3‐year‐old full‐size individual), 2.0 (3‐year‐old jack), and 2.1 (4‐year‐old full‐size individual). The number before the decimal place represents the number of years spent rearing in freshwater and the number after the decimal represents the number of years at sea.

**TABLE 1:** Auke Creek Coho Salmon life stages.

Type	F	W	Sp	Su	F	W	Sp	Su	F	W	Sp	Su	F	W	Sp	Su	F	
1.0 Jack	Egg		Hatch				Smolt		Spawn	2 year								
2.0 Jack	Egg		Hatch								Smolt		Spawn	3 year				
1.1 Full‐size	Egg		Hatch				Smolt						Spawn	3 year				
2.1 Full‐size	Egg		Hatch								Smolt						Spawn	4 year

*Note*: Labels and cell shading denote durations of life stages. Eggs are laid in the gravel in the fall and hatch the following spring. All individuals spend the next 1 or 2 years rearing in freshwater. After smolting, jacks spend 6 months at sea before returning to spawn at age 2 or 3 years old. Full‐size individuals spend 1 full year at sea before returning to spawn at age 3 or 4 years old.

### 2.2 Genotyping and parentage assignment

Genotyping and parentage assignment methods were the same as those presented in King et al. (2023). In brief, we employed a contract lab (GTseek LLC) to extract and sequence tissue samples of all returning individuals from 2009 to 2019 using the “genotyping‐in‐thousands” (GTseek LLC) protocol (Campbell et al., 2015). The single‐nucleotide polymorphism (SNP) panel used, composed of 259 loci, was developed specifically for Coho Salmon parentage by the Columbia River Inter‐Tribal Fisheries Commission (Hess et al., 2016).

We used the program FRANz (Riester et al., 2009) to assign parentage for adult offspring from each individual return year from 2013 to 2019. We constrained the set of possible parents for each year to fish that returned 2–4 years prior. Sex was not used in parentage assignment due to uncertainty in field identification. For each FRANz run we used the following parameters: *N*_max_ was calculated by multiplying the number of potential parents, as enumerated at the weir, by (1.1)/2 (half of the potential parents with a 10% buffer); genotyping error rate was assumed to be 0.01 (default FRANz value); the allowed maximum number of mismatching alleles was five for dyads and seven for triads; and the minimum loci typed per individual was 150 (out of 251). Parentage assignments with posterior probability <0.9 were discarded. We expected assignment error to be small because we genotyped such a large proportion of the population and the panel had 122 loci with minor allele frequency >0.25. We determined whether fish captured in 2013–2015 with intact adipose fins were likely strays (i.e., individuals that assigned with high confidence to no Auke Creek parents) or were unmarked locals (i.e., individuals that assigned with high confidence to at least one parent from Auke Creek). We then quantified the percent of returning adults from stray parents that spawned in 2013–2015. For additional details, see King et al. (2023).

### 2.3 Demographic estimate of *N*_b_ and *N*_e_

Parentage assignment allowed direct calculation of the inbreeding effective number of breeders (*N*_bD_) in the population each year, and from this demographic information an inbreeding effective population size (*N*_eD_) was calculated for each generation (for full notation see Table 2). *N*_e_ estimates apply over a generation (4 years for Auke Creek Coho Salmon), while *N*_b_ estimates are annual. First, we calculated *N*_bD_ of each sex for each return year from 2013 to 2019 using population size (*N*), mean number of offspring ($\bar{k}$), and variance in the number of offspring per individual (*V*_*k*_) with the following equation (Crow & Denniston, 1988; Crow & Kimura, 1970):

Nbifemale≈k¯fNf−1k¯f−1+Vkfk¯f,Nbimale≈k¯mNm−1k¯m−1+Vkmk¯m

**TABLE 2:** Notation used to describe demographic and genetic parameters in this paper.

Term	Definition
*N*	Census population size
*N*_e_	Effective population size. Applies to an entire generation
*N*_b_	Effective number of breeders. Applies to a single reproductive cycle, which is 1 year for Pacific salmon
*N*_eD_	Effective population size calculated using demographic information
*N*_bD_	Effective number of breeders calculated using demographic information
*N*_bDf_	Effective number of female breeders calculated using demographic information
*N*_bDm_	Effective number of male breeders calculated using demographic information
*N*_eLD_	Effective population size calculated using the linkage disequilibrium method
*N*_bLD_	Effective number of breeders calculated using the linkage disequilibrium method
*N*_bS_	Effective number of breeders calculated using the SALMONNb method
*N*_eLT_	Effective population size calculated using the life table method
*N*_bLT_	Effective number of breeders calculated using the life table method
*g*	Generation length
$\bar{k}$	Mean number of offspring produced per individual
*V*_*k*_	Variance in number of offspring produced per individual

After this, we calculated the net *N*_bD_ for each year [*i*] using the male and female effective number of breeders with the following equation (Crow & Kimura, 1970; Wright, 1931):

$$N_{b\left[i\right]}=\frac{4\left(N_{b\left[i\right]}^{\text{female}}\times N_{b\left[i\right]}^{\text{male}}\right)}{\left(N_{b\left[i\right]}^{\text{female}}+N_{b\left[i\right]}^{\text{male}}\right)}$$

Then *N*_eD_ values for different generations were estimated using *N*_bD_ estimates from the corresponding years in each generation:

NeD=1∑Xi2Nbi

where *X*_*i*_ is the proportional contribution of breeders from year *i* to the next generation (Ryman & Laikre, 1991).

Coho Salmon return to Auke Creek up to 4 years after being spawned, so for each generation we calculated four *N*_eD_ values using overlapping *N*_bD_ values with a sliding scale of 4 years over 11 years (2009–2012, 2010–2013, 2011–2014, and 2012–2015). Individuals that were not successfully genotyped were included in the calculations of sex‐specific *N*_b_ and were assumed to have the same mean and variance in reproductive success as those successfully genotyped of the same sex. In 2010, of the 816 individuals that returned, 109 were not assigned a sex. In each of 400 iterations, we randomly assigned the unsexed fish a sex (given a 50:50 sex ratio) and then calculated *N*_bDf_, *N*_bDm_, and *N*_bD_. To estimate 2010 *N*_bD,_ we used the mean *N*_bD_ from all 400 iterations. Estimates of *N*_bD_ for 2010 and *N*_e_ for generations including 2010 using a 50:50 sex ratio were almost identical to using the average sex ratio (58:42) from 2009, 2011, 2012, 2013, 2014, and 2015.

To examine the sizes of *N*_bD_ and *N*_eD_ relative to the population size, we calculated two ratios. In *N*_bD_/*N*, *N* represents the number of individuals returning to spawn in a single year, whereas *N* in *N*_eD_/*N* represents the total number of individuals returning over the entire generation (which includes multiple spawning/return years).

To investigate the specific contribution of jacks to *N*_bD_, we also calculated *N*_bD_ using the same dataset but removing all jack parents and their offspring (after Perrier et al., 2014; Saura et al., 2008). Jack contribution was defined in the following way:

$$\text{JackContribution}=100\times \frac{N_{\text{bDwithjacks}}-N_{\text{bDwithoutjacks}}}{N_{\text{bDwithjacks}}}$$

Jack contribution represents the percent difference in annual estimates of *N*_bD_ when jacks are excluded from calculations. Positive values would indicate that the presence of jacks increases *N*_bD_.

We made three choices when excluding jacks from the dataset. First, we excluded the year 2010, because there was a high proportion of unidentified sex/type (i.e., jack vs. regular male) samples that year and this analysis relied on comparing totals of full‐size males and jacks in the population. Second, we excluded all parents of unidentified sex/type across all years. Third, mean and variance in reproductive success were calculated with the number of retained individuals that were successfully genotyped. We assumed that the individuals not successfully genotyped had the same reproductive success as those that were successfully genotyped. In the calculation of *N*_bD_, the total N included all individuals (genotyped and not genotyped). We expect the bias introduced by calculating *N*_bD_ this way to be small given the high rate of genotyping success (92% of all individuals from 2009 to 2019) we achieved.

### 2.4 Genetic estimates of *N*_b_ and *N*_e_

*N*_b_ was estimated using the linkage disequilibrium (LD) method (*N*_bLD_) (Hill, 1981; Waples, 2006b; Waples & Do, 2008) as implemented in NeEstimator V2.1 (Do et al., 2014) and the temporal method of Waples et al. (2007) in the program SALMONNb (*N*_bS_). For *N*_bLD_ and *N*_bS_, we analyzed individual cohorts (individuals born in the same year) for each year 2009 to 2015. Individuals were grouped into cohort years using parentage‐based aging. Bias in the estimates of *N*_bLD_ was corrected using the haploid number of chromosomes (Waples et al., 2016), which for Coho Salmon is 30 (Uyeno, 1972).

### 2.5 Using life tables to estimate *N*_e_

Life tables describe the abundance, survivorship rate, and offspring produced for each age group in a population or species. Life tables can be used to calculate the number of offspring produced from each age group, based on the proportions of individuals at each age and the mean and variance in number of offspring of individuals in each age group (Waples et al., 2011). For iteroparous species, individuals can reproduce at multiple ages and each row of the life table represents each year of an individual's life. But in semelparous species, all individuals die after reproducing, and in this case, the life table describes individuals that return to spawn in a single year and there is a row for each age individuals return to spawn. For example, Auke Creek Coho Salmon females spawn at ages 3 or 4, so the female life table has a row for age 3 individuals and age 4 individuals.

We created one composite life table for each sex using either all males or females from 2013 to 2015. These years were chosen because they were the only years for the study where we had parentage‐based age estimates (needed for separating the population into age classes) and samples of all possible returning offspring from these individuals (needed to calculate reproductive success of the individuals). To create each life table, we first determined the number of individuals in each age class. For males, we used the age classes 1.0, 1.1–2.0, and 2.1, where the number before the decimal denotes the inferred number of years an individual spent rearing in freshwater and the number after the decimal denotes the number of years an individual spent in the ocean before returning to spawn. The corresponding ages of those age classes are 2, 3, and 4 years old, respectively. Freshwater age was deduced by subtracting the saltwater age (known based on male type) from the total parentage‐based age of an individual. For females, we used the age classes 1.1 and 2.1 (age 3 and 4, respectively) because all females spend a full year at sea.

For each age class within the group, we determined the number of individuals, mean number of offspring produced, and variance in number of offspring produced. We scaled the mean and variance of all age classes to a stable population (*k* = 2). With the age‐specific information, we calculated the total number of offspring produced, the net variance in reproductive success for the whole table, the effective number of breeders, and the generation length (*g*). Each sex‐specific g was calculated as the sum of each age class multiplied by their relative contribution of offspring. We then combined the male and female table information to determine overall *N*_bLT_, *g*, and *N*_eLT_ (*N*_eLT_ = *g* × *N*_bLT_).

## 3 RESULTS

### 3.1 Demographic estimate for Auke Creek Coho

We created a multi‐year, multi‐generation pedigree from genotypes collected from Coho Salmon captured at the Auke Creek weir in 2009–2019. These data allowed us to assign offspring to parents, to determine the reproductive success of jacks and full‐sized adults, and to determine the proportion of offspring produced by immigrants into this population from elsewhere. The mean annual jack contribution to *N*_bD_ was 12.9% (SE = 3.8%), and closer inspection of their contribution to *N*_bD_ shows they increased *N*_bD_ via male‐specific values, *N*_bDm_, with little impact on female‐specific values, *N*_bDf_ (Table 3). The mean annual jack contribution to *N*_bDm_ was 23.9% (SE = 6.3%), and jacks increased *N*_bDm_ in every year except 2012. In contrast, the mean annual jack contribution to *N*_bDf_ was 0.7% (SE = 2%). In two of 6 years, *N*_bDf_ without jacks was greater than *N*_bDf_ with jacks because excluding jacks decreased the female variance in reproductive success in those years which increased *N*_bDf_. In 2012, *N*_bDf_ without jacks was larger than *N*_bDf_ with jacks and *N*_bDm_ without jacks did not change, so the overall *N*_bD_ without jacks (174) was larger than *N*_bD_ with jacks (170) and jack contribution was negative (−2.9%). *N*_bD_/*N* did not notably change when jacks were excluded because jacks affect both the numerator and denominator of this ratio.

**TABLE 3:** Demographic information and effective number of breeders (*N*_b_) calculated using the demographic method (*N*_bD_) for Auke Creek Coho Salmon.

Year	Number of returning individuals				Sex ratio (M/F)	Jack frequency	*N*_b_						
							Female		Male		Total		Percent jack contribution
	Female	Full‐size male	Jack male	Total			All	No jacks	All	No jacks	All	No jacks	
2009	178	150	126	454	1.55	0.46	64	62	78	54	141	116	17.7
2011	282	215	377	874	2.1	0.64	54	53	83	46	130	99	23.8
2012	467	369	122	958	1.05	0.25	77	82	94	94	170	175	−2.9
2013	406	332	211	949	1.34	0.39	107	106	117	81	224	183	18.3
2014	760	768	304	1832	1.41	0.28	168	173	260	197	407	368	9.6
2015	293	287	76	656	1.24	0.21	50	46	60	52	109	97	11.0

*Note*: Number of returning individuals in this table excludes individuals with unknown sex and return year. Jack frequency is the ratio of jacks to all males. *N*_b_ values under “All” were calculated using all individuals and values under “No jacks” were calculated with all jacks and offspring of jacks removed. Percent jack contribution is the percent difference between *N*_b_ with and without jacks included in *N*_b._

**FIGURE 1:**
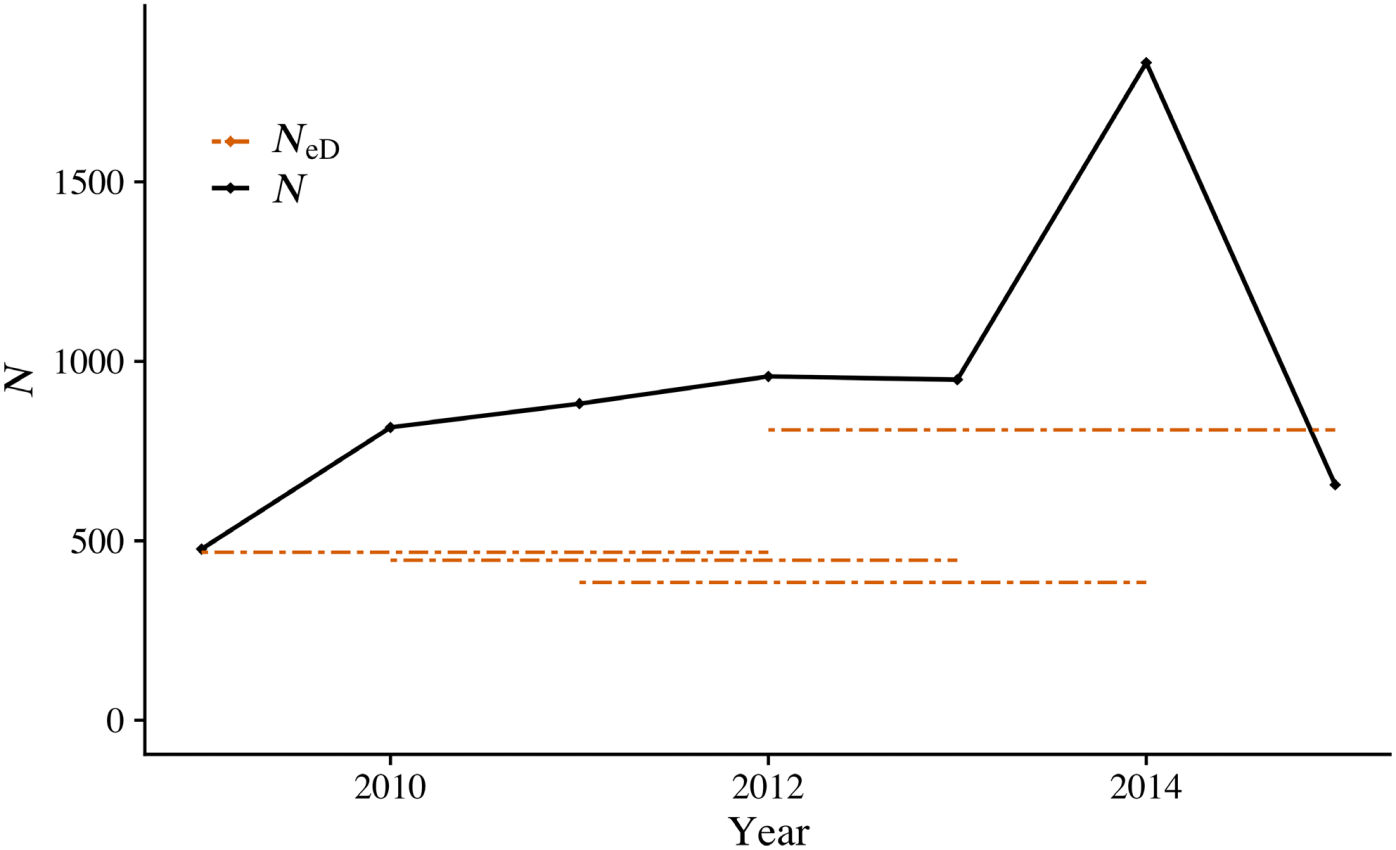
Auke Creek Coho Salmon population size including unknown sex/type adult individuals (*N*) and effective population size (*N*_eD_) for four overlapping generations calculated using yearly effective number of breeders values and the proportional contribution of offspring from each year.

*N*_bDf_ each year ranged between 50 and 168, while *N*_bDm_ ranged from 60 to 260. In all 6 years analyzed, *N*_bDm_ exceeded *N*_bDf_. Combined, the total *N*_bD_ ranged from 109 to 407 (Table 3). Over the time series, *N*_bD_ relative to the census size each year declined rapidly, increased in 2012 and 2013, and then decreased (Figure S1). *N*_bD_/*N* oscillated between 0.15 and 0.3 from 2009 to 2015. Assuming 4 years per generation, the maximum *N*_eD_ was 812 and the minimum was 385 (Figure 1 and Table S1). *N*_eD_/*N* for the four 4‐year periods was 0.16, 0.13, 0.08, and 0.18 (Table S1).

Between 0.84% and 9.76% of returning adults each year did not have a fin clip, implying that they may be strays into Auke Creek. Of these, 168 (63.9%) were likely strays (assigned with high confidence to no Auke Creek parents). Most strays were not successful at producing offspring, but strays produced 4.5% of all offspring from 2013 to 2015 that returned to Auke Creek as adults in subsequent years (through 2019).

### 3.2 Genetic estimates of *N*_b_ and *N*_e_

**FIGURE 2:**
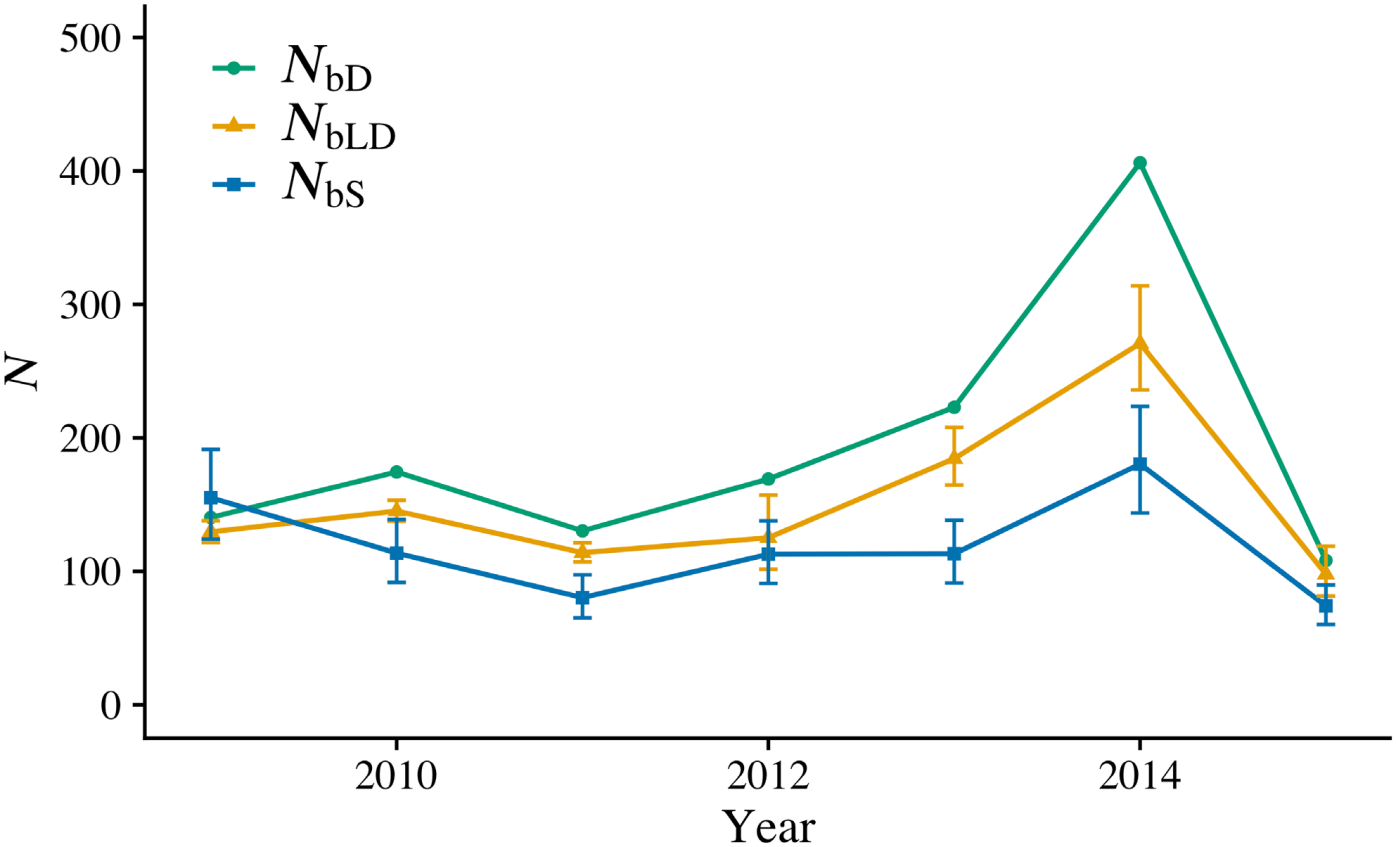
Inbreeding effective number of breeders over time using the demographic method (*N*_bD_), linkage disequilibrium method (*N*_bLD_), and SALMONNb method (*N*_bS_) for Auke Creek Coho Salmon from 2009 to 2015. 95% CIs are included (for *N*_bLD_ and *N*_bS_).

*N*_bLD_ (range: 98–271) and *N*_bS_ (range: 74–180) followed the same overall pattern of *N*_bD_ (Figure 2 and Table S2): a gradual increase from 2009 to 2014, then a steeper decline to 2015. *N*_bD_, *N*_bLD_, and *N*_bS_ are consistent because these estimates are all closely tied to inbreeding effective size and apply to the year in which the parents of genotyped individuals reproduced (Waples, 2005). The annual *N*_bD_, *N*_bLD_, and *N*_bS_ values contributed to per generation estimates of *N*_eD_ = 613, *N*_eLD_ = 514, and *N*_eS_ = 404.

### 3.3 Using life tables to estimate *N*_e_

The composite life tables for males and females created from our demographic and reproductive success data from 2013 to 2015 revealed that the overall generation length was 3.72 years (Male: 3.61, Female: 3.84), *N*_bLT_ was 564 (Male: 306, Female: 261), and *N*_eLT_ was 2099 (Table 4). Full‐size males contributed the most offspring, but the contribution from jacks was still substantial (jacks produced 17.3% of offspring in the table). Most of the jack contribution was from age‐3 jacks, as age‐2 jacks were less common (5.5% of jacks were age‐2). Overall, jacks had smaller mean and variance in reproductive success (Age 2: *b*_*x*_ = 0.19 *V*_*x*_ = 0.32; Age 3: *b*_*x*_ = 0.26 *V*_*x*_ = 0.53) than full‐size males (Age 3: *b*_*x*_ = 0.5 *V*_*x*_ = 1.24, Age 4: *b*_*x*_ = 0.5 *V*_*x*_ = 1.58), but similar mean‐adjusted variance. Age‐4 females had similar reproductive success (*b*_*x*_ = 0.55) to age‐3 females (*b*_*x*_ = 0.53), but their higher abundance resulted in a greater contribution of offspring than age‐3 females.

**TABLE 4:** Female and male composite life tables for Auke Creek Coho Salmon, created using individuals of known age and genotype that returned from 2013 to 2015.

Sex	Age class	Age	*N*_x_	*N*_x_/*N*	*b*_x_	*B*_x_	*V*_x_	*b*'_x_	*B*'_x_	*V*'_x_
Female	1.1	3	209	0.16	0.53	111	1.95	2.21	463	26.92
	2.1	4	1061	0.84	0.55	585	1.66	2.3	2439	21.55
Male	1.0	2	26	0.02	0.19	5	0.32	0.8	21	3.02
	1.1	3	297	0.18	0.5	149	1.24	2.08	618	14.72
	2.0	3	443	0.27	0.26	116	0.53	1.09	481	5.75
	2.1	4	866	0.53	0.5	430	1.58	2.06	1783	20.72

*Note*: All age‐3 males are pooled together (jacks and full‐size males). Abundance (*N*_x_), proportion (*N*_x_/*N*), mean number of offspring produced (*b*_x_), total number of offspring produced (*B*_x_), and variance in number of offspring produced (*V*_x_) are shown for each age class. For a stable population, the mean number of offspring (*b*'_x_), total number of offspring produced (*B*'_x_), and variance in number of offspring (*V*'_x_) are noted with the prime symbol (’).

## 4 DISCUSSION

Our study yielded several important insights into the influence of life‐history variation on *N*_e_. First, jacks made a substantial contribution to *N*_e_, but their contribution varied widely across years. Jack Coho Salmon in Auke Creek made up a large proportion of the spawning males in some years and contributed a significant number of offspring. Excluding jacks from *N*_e_ calculations could bias these estimates, resulting in faulty inferences about the reproductive contributions of full‐size males, and cause incorrect conclusions about the status of populations being monitored using *N*_e_. Second, different methods to estimate *N*_b_ produced similar results and reflected changes in adult abundance over time, despite variation in the *N*_b_/*N* ratio. We also detected a small, but potentially important genetic contribution of immigrants into this population.

### 4.1 Effect of jacks on *N*_e_

The presence of jacks in this population generally increased *N*_b_ (male and overall), but the magnitude of the contribution was highly variable from year to year. Removing jacks decreased the number of males and typically increased the variance in reproductive success which lowered *N*_bm_. Reductions in *N*_bD_ were primarily caused by the smaller numbers of individuals because removing jacks had little effect on *N*_b_/*N* (the difference between the two estimates was consistently <0.05). Similarly, a study on Atlantic Salmon found that including mature male parr increased *N*_e_ without changing *N*_e_/*N* (Saura et al., 2008). Although there is no consensus on the typical range of *N*_e_/*N* values for natural populations of plants and animals (Waples, 2002), the *N*_eD_/*N* for Auke Creek Coho Salmon (0.08–0.18) is comparable to the values reported for a wide range of species. Frankham (1995) first reported that *N*_e_/*N* averaged ~0.11 over 100 species and a more recent review by Palstra and Ruzzante (2008) found a median *N*_e_/*N* of 0.14.

We found little effect of removing jacks on *N*_bDf_, which aligns with a study on Atlantic Salmon that found that mature male parr exclusion or inclusion had a negligible impact on female *N*_e_ (Perrier et al., 2014). We did observe 1 year when *N*_bDf_ was larger without jacks because removing jacks decreased the variance in female reproductive success and *N*_bDm_ did not change after removing jacks, so overall *N*_bD_ without jacks was slightly larger than with jacks. The lack of influence on *N*_bDf_ suggests that spawning success of females in this population is not male limited. Females are the less abundant sex and 88% of females with at least one identified mate had one or more full‐size male mates.

### 4.2 Comparison across estimates

All *N*_b_ estimates showed a similar temporal pattern and reflected the dynamics in the number of returning adults. While the *N*_bLD_ values were slightly lower than the *N*_bD_ values, they followed the same trend for the entire series, which supports the potential of *N*_b_ estimates to serve as a population monitoring tool (Charlier et al., 2012; Luikart et al., 2021; Tallmon et al., 2010). Other studies have also found close relationships between demographic and LD estimates of *N*_b_, yet comparisons on this scale are still relatively rare. A study of the impact of Atlantic Salmon mature male parr on N_b_ found that the demographic estimate (220) and LD estimate (198) were very similar (Perrier et al., 2014). Serbezov et al. (2012) found similar demographic *N*_b_ (40) and LD *N*_b_ (53) in a population of Brown Trout *Salmo trutta*.

*N*_e_ from the demographic method (*N*_eD_ = 613) was larger than the LD method (*N*_eLD_ = 514). The genetic estimates may be lower than the demographic estimate because of immigration effects from fluctuations in population size across generations, or physical linkage of marker loci. The legacy effects of immigration, sweepstake events, and past small population sizes can carry over among years and affect genetic estimates of *N*_e_ (Ryman et al., 2014; Waples & England, 2011). The demographic estimate is not as impacted by small population sizes as the genetic estimates because there are no population genetic processes, beyond estimating *V*_*k*_ and *k* from parentage, when calculating *N*_eD_ from demographic information.

The amount of migration into the population and the reproductive success of those immigrants may have influenced our genetic estimates of effective population size because individuals identified as likely strays contributed 4.5% of offspring produced 2013–2015. The presence of strays in the Auke Creek population could explain why *N*_bLD_ is slightly lower than *N*_bD_ and why *N*_eLD_ was smaller than *N*_eD_, but the influences of immigration on *N*_b_ and *N*_e_ are complex. Sampling individuals from divergent subpopulations or following pulses of migration can decrease *N*_bLD_ estimates (Waples **&** England, 2011; Whiteley et al., 2017). Ryman et al. (2019) found that even one migrant per generation can cause differences among the *N*_e_ estimates of commonly used methods in a modeled population with discrete generations. Identifying the source of strays and the size, structure, and migration rates of regional subpopulations of Coho Salmon in Southeast Alaska will improve our understanding of how immigration affects our various *N*_b_ and *N*_e_ estimates.

### 4.3 Conservation and evolutionary implications

Monitoring *N*_e_ of Auke Creek Coho Salmon is important because returns declined to an all‐time low (since weir operation started in 1980) of 159 individuals in 2018, and it remains unclear whether the trend will continue. There was a slight increase in 2019 (457), 2020 (309), and 2021 (365), but these years were still low compared to the long‐term average (prior to 2014) return of 924 total adults and 675 full‐size adults. The escapement goal set for Auke Creek Coho Salmon (jacks excluded) is 200–500 (Clark et al., 1994). From 2009 to 2017, 2019, and 2021, the escapement either met or exceeded this goal, but not in 2018 and 2020. Monitoring the contribution of each life‐history type in this population and others will provide insight into the population dynamics of declining stocks, which may facilitate conservation efforts for vulnerable populations.

Understanding the genetic contributions of different life‐history types provides insight into how populations may respond to stressors such as climate change and habitat degradation. For example, different age‐classes can experience different magnitudes and direction of selection on traits like body size (Ulaski et al., 2020; Watters et al., 2003). Coho Salmon have less variability in total age than Chinook and Sockeye Salmon, potentially making them more vulnerable to longer lasting and more frequent natural and anthropogenic disturbances (Waples et al., 2008). Jacks, by spending less time at sea, might help compensate for low full‐size male returns in periods of high marine mortality. There is accumulating evidence that life‐history variation provides resilience and stability in natural populations (Moore et al., 2014; Munsch et al., 2022; Watters et al., 2003), but this life‐history variation affects average reproductive success and its variance, further underscoring the need to monitor *N*_b_ and *N*_e_ in populations of conservation concern. The contribution of multiple cohorts to an individual return year reduces the effects of variable recruitment among years. Perrier et al. (2014) found that Atlantic Salmon mature male parr increased *N*_b_ and allelic richness and hypothesized that the presence of mature male parr may compensate for low amounts of returning full‐size males and potentially buffer declines in *N*_e_. Jacks, by spending less time at sea, might help compensate for low full‐size male returns in periods of high marine mortality. In this population, jacks increased *N*_b_ in most years.

Although our results show jacks tend to increase *N*_b_, the ability of jacks to bolster *N*_e_ across a range of abundances depends upon complex relationships between *N*, *N*_b_, and the frequency of jacks relative to full‐size males. Fluctuations in annual recruitment can result in jacks from larger cohorts mating among full‐size males from smaller cohorts, which increases jack representation (DeFilippo et al., 2019). Harvest of full‐size males may also increase the frequency of jacks (Young et al., 2020). On an individual basis, jacks tend to have lower reproductive success at higher jack frequencies (Berejikian et al., 2010), which may attenuate their contribution to *N*_b_ and *N*_e_ when relatively abundant. We observed the lowest *N*_b_/*N* (0.15 and 0.17) at high (0.64) and low (0.21) jack frequencies. The highest *N*_b_/*N* (0.3) occurred at a moderate jack frequency (0.46). The additional study of the relationship between jack frequency and the magnitude of influence of jacks on *N*_b_/*N* would help to quantify the value of life‐history variants such as jacks as a buffer for *N*_b_ and *N*_e_ over a range of population sizes, including small populations of conservation concern.

From a conservation perspective, the *N*_e_ and immigration data suggest it is important to consider Auke Creek Coho in a regional context. The *N*_e_ values we observed of at least a few hundred per generation suggest that a loss of genetic variation is not a short‐term threat to Auke Creek Coho Salmon. We also identified that strays (immigrants) contributed 5% of returning adult offspring. Strays will add genetic variation that can be adaptive, maladaptive, or neutral, but we expect local genetic variation will be maintained at higher levels than predicted strictly from the local *N*_e_ estimates above to reflect the *N*_e_ of populations in the region linked by migrants (Palstra & Ruzzante, 2008; Ryman et al., 2019). Because this Coho Salmon population has rapidly shifted its run timing over the past four decades, presumably in response to changing environmental conditions (Kovach et al., 2013), it would be helpful to know whether continued straying will aid this population's future adaptation. It is clear from our results that valuable management and evolutionary insights can be gained from using multi‐generation genetic data to infer the contributions of life‐history variants, such as small males and strays, frequently ignored in studies of wild populations.

## CONFLICT OF INTEREST STATEMENT

We declare no conflicts of interest.

## Supporting information


Figure S1:

## Data Availability

The data and scripts that support the findings of this study are openly available in Dryad at https://doi.org/10.5061/dryad.2280gb5z6. The raw data that supports the findings of this study are available in the supplementary material of King et al. 2023 at http://doi.org/10.1098/rsos.221271.
