# Supplementary material for: Alternative life‐history strategy contributions to effective population size in a naturally spawning salmon population
Source: Evol Appl. 2023 Jul 14;16(8):1472–82. doi: 10.1111/eva.13580 (PMC10445090; doi:10.1111/eva.13580)

**Supplementary Material**


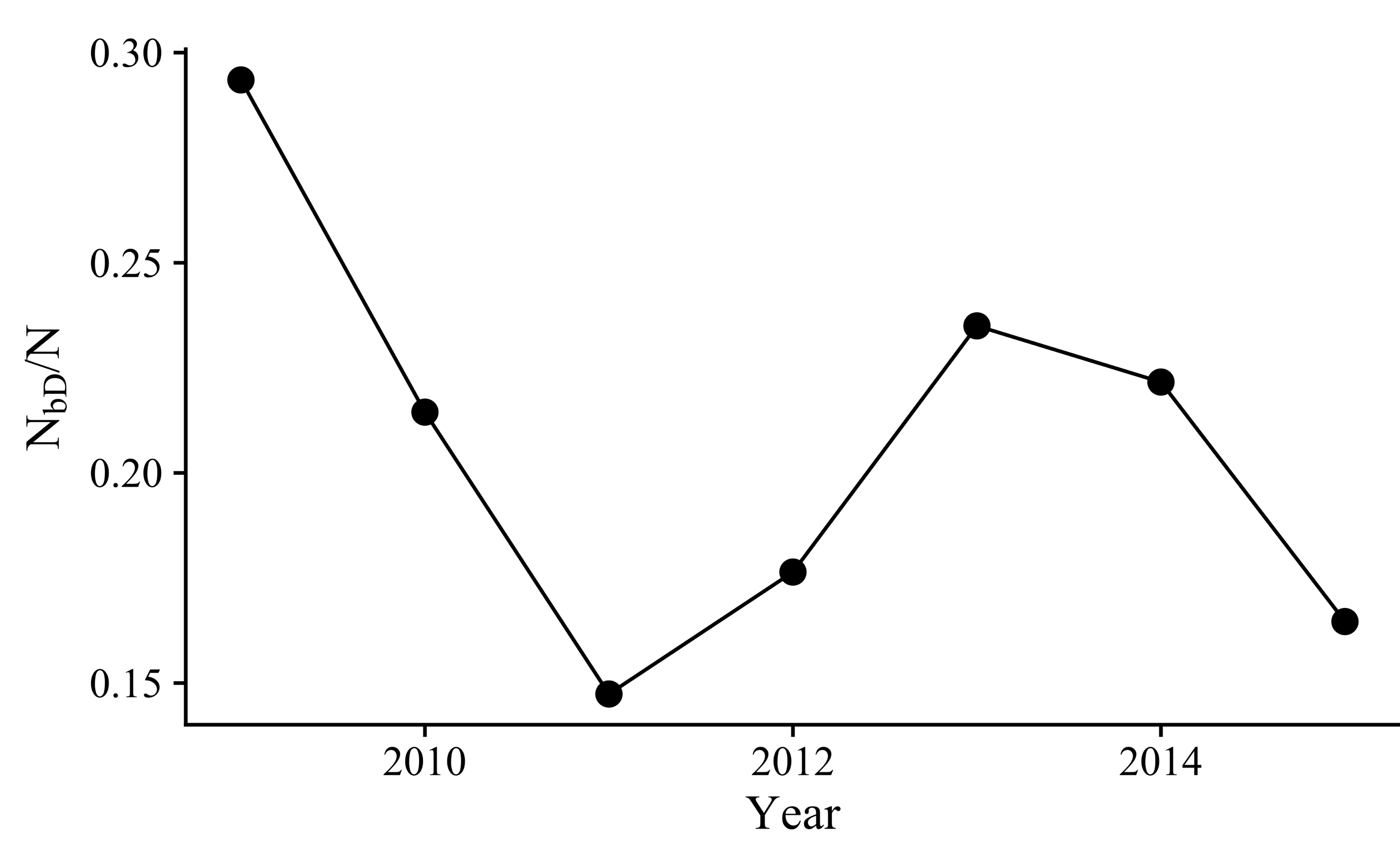


Figure S1: The ratio of effective number of breeders calculated using demographic information to population size of Auke Creek Coho Salmon, 2009-2015.

Table S1: Auke Creek Coho Salmon effective population size (N_e_) calculated using yearly effective number of breeders (N_b_) values and the proportional contribution of offspring from each year. Mean yearly N is the average number of the individuals returning each year in the generation and Total N is all returning individuals in the generation. These census values do not include individuals with unknown sex/type.


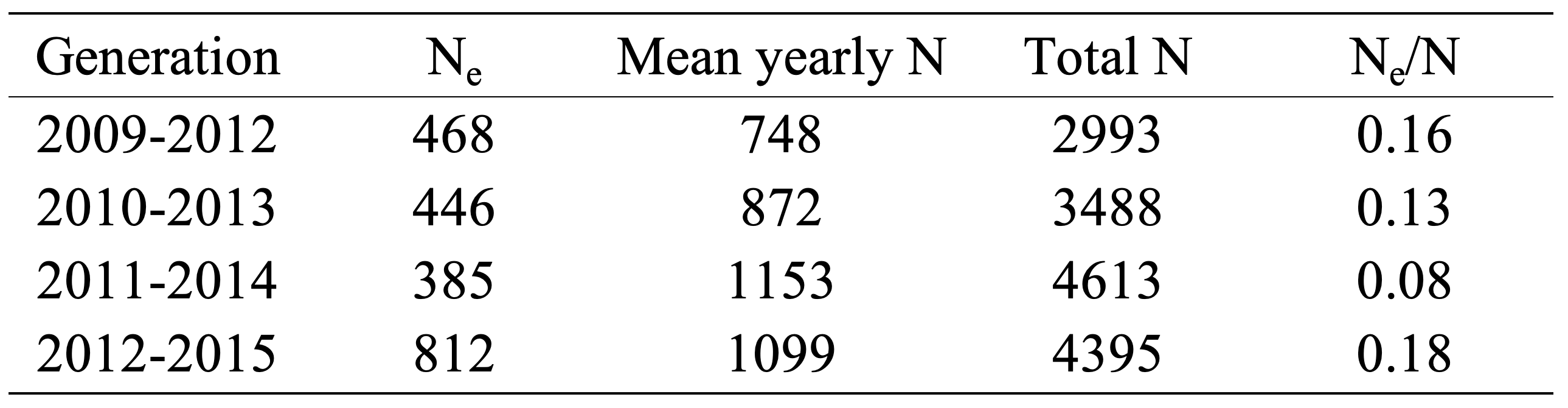


Table S2: Inbreeding effective number of breeders (N_b_) and ratio of N_b_ to number of returning individuals (N) using the demographic method (N_bD_), linkage disequilibrium (N_bLD_), and SALMONNb method (N_bS_) for Auke Creek Coho Salmon from 2009 to 2015. 95% Confidence intervals are included for N_bLD_ and N_bS_. N includes individuals that were not successfully genotyped.


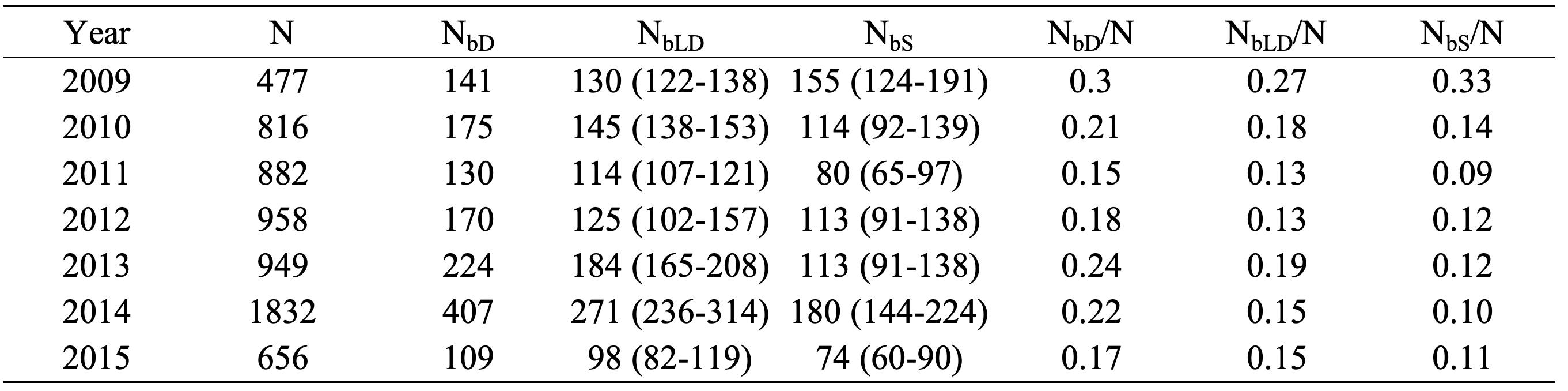

Supplement: Supplementary file 1 — Figure S1: [file EVA-16-1472-s001.docx]
